# The Public Health Risks of Colistin Resistance in Dogs and Cats: A One Health Perspective Review

**DOI:** 10.3390/antibiotics14121213

**Published:** 2025-12-02

**Authors:** Juliana Menezes, Laura Fernandes, Cátia Marques, Constança Pomba

**Affiliations:** 1 I-MVET—Research in Veterinary Medicine, Faculty of Veterinary Medicine, Lusófona University-Lisbon University Centre, 1749-024 Lisbon, Portugal; p6194@ulusofona.pt; 2 Associate Laboratory for Animal and Veterinary Sciences (AL4AnimalS), 1300-477 Lisbon, Portugal; lauraf@fmv.ulisboa.pt; 3 Centre for Interdisciplinary Research in Animal Health (CIISA), Faculty of Veterinary Medicine, University of Lisbon, 1300-477 Lisbon, Portugal; 4 CECAV—Animal and Veterinary Research Center, Faculty of Veterinary Medicine, Lusófona University-Lisbon University Centre, 1749-024 Lisbon, Portugal

**Keywords:** *mcr* genes, companion animals, antimicrobial resistance, zoonotic transmission, plasmid-mediated dissemination, animal–human sharing, transmission pathways

## Abstract

Colistin, a polymyxin antibiotic considered a last-line treatment for multidrug-resistant Gram-negative infections, has been widely used in livestock, promoting resistance in bacterial populations that can disseminate through the environment. Although rarely used in companion animals, dogs and cats can acquire and spread colistin-resistant strains through shared environments, acting as potential reservoirs of resistance. Reliable detection of resistant strains remains challenging due to technical limitations of routine susceptibility tests. Despite these constraints, epidemiological studies demonstrate the global presence of colistin-resistant bacteria in companion animals, with multiple plasmid-mediated colistin-resistant genes (*mcr*) identified in different bacteria species. Evidence of clonal and plasmid-mediated sharing of resistant strains between companion animals, humans, and, in some cases, food-producing animals highlights the complex and multidirectional nature of transmission. Although the directionality of transmission remains difficult to establish, the detection of colistin-resistant bacteria in companion animals is concerning. Addressing this challenge requires a One Health approach, integrating coordinated surveillance and infection and control measures in veterinary practices to safeguard the effectiveness of this critical last-resort antibiotic. This review summarizes current knowledge on colistin resistance mechanisms, diagnostic challenges, epidemiology, and the potential for interhost transmission, highlighting the role of dogs and cats as potential reservoirs of colistin resistance.

## 1. Introduction

Antimicrobial resistance (AMR) has emerged as a pressing global health issue with far-reaching consequences for society, including increasing mortality and morbidity rates, as well as greater utilization of healthcare resources [1]. It is currently estimated that antibiotic resistant infections are responsible for around 1.27 million deaths each year globally. This number is projected to rise to 10 million annual deaths by 2050 if current trends persist [2]. A key driver of this growing threat is the spread of resistance genes, often facilitated by mobile genetic elements (MGEs) such as plasmids, which enable the rapid transfer of resistance traits between bacteria [3]. Moreover, the accumulation of multiple resistance markers within a single pathogen [4,5], raises serious concerns about the emergence of multidrug-resistant (MDR), extensively drug-resistant (XDR), and even pandrug-resistant (PDR) organisms.

Originally introduced in the late 1940s, colistin was later abandoned for decades due to its nephrotoxicity and neurotoxicity [6,7]. It was derived from the soil bacterium *Paenibacillus polymyxa* subsp. *colistinus* [6], and is particularly effective against a range of Enterobacterales bacteria, including *Escherichia coli*, *Enterobacter* spp., *Klebsiella* spp., *Citrobacter* spp., *Salmonella* spp., and *Shigella* spp. [7,8,9]. Additionally, it demonstrates a significant activity against common non-fermenting Gram-negative pathogens, such as *Acinetobacter baumannii*, *Pseudomonas aeruginosa*, and *Stenotrophomonas maltophilia* [8]. In contrast, certain bacterial species display intrinsic resistance to polymyxins, including *Chromobacterium* spp., *Brucella* spp., *Burkholderia cepacia*, *Edwardsiella* spp., *Campylobacter* spp., *Morganella morganii*, *Legionella* spp., *Proteus* spp., *Providencia* spp., *Pseudomonas mallei*, *Serratia marcescens* and *Vibrio cholerae*. Moreover, polymyxins generally lack activity against Gram-negative cocci, as well as against Gram-positive and anaerobic bacteria [8].

Colistin belongs to the polymyxin class, which consists of cyclic, non-ribosomal oligopeptide antimicrobials. This class harbors five chemically distinct compounds, polymyxins A, B, C, D, and E (colistin), although only polymyxin B and colistin are commonly used in medical treatments [7]. Colistin differs from polymyxin B by a single amino acid in the peptide ring (D-leucine replaces D-phenylalanine) [7].

Amid the global rise in MDR infections, therapeutic options have become increasingly limited [10], and, in light of the scarcity of new antibiotics effective against certain MDR bacteria, namely carbapenem-resistant Gram-negatives, colistin has regained relevance as a last-resort antimicrobial in human medicine [11]. In veterinary practice, particularly in food-producing animals, colistin has been employed for the prevention and treatment of Enterobacterales infections [11]. However, and reflecting its renewed critical role, the World Health Organization has listed colistin among the highest priority critically important antimicrobials (HPCIAs), which also include extended-spectrum cephalosporins, quinolones, macrolides, and glycopeptides, advising a limitation of its use in veterinary medicine, underscoring the need for prudent and controlled use to avoid further resistance dissemination [10].

Unfortunately, there has been a gradual increase in colistin-resistant bacteria in recent years, reported across humans, food-producing animals, companion animals, wildlife, and various environmental settings worldwide [12,13]. The presence of colistin-resistant bacteria in companion animals is particularly concerning given the close contact with humans, which may facilitate zoonotic transmission. Such sharing of resistant strains has already been documented in cohabiting dogs, cats and humans [14,15].

This review aims to synthesize current knowledge on colistin use and resistance in companion animals, evaluate the potential public health implications, and discuss strategies for mitigating the spread of resistance within the One Health framework.

## 2. The Use of Colistin in Veterinary Medicine

Colistin was initially introduced into human medicine during the 1950s, following its intravenous approval by the US Food and Drug Administration in 1959 for the management of Gram-negative bacterial infections, including urinary tract infections and infectious diarrhea [16]. Over subsequent decades, polymyxins were also applied topically, particularly in ophthalmic and otic preparations, and for selective digestive decontamination protocols [8]. Their systemic use, however, was curtailed for many years because of well-documented neurotoxic and nephrotoxic side effects [7]. One notable exception was in patients with cystic fibrosis, who continued to benefit from systemic or inhaled colistin to manage chronic pulmonary infections [7,8,9]. More recently, the global surge of multidrug-resistant Gram-negative pathogens has renewed interest in parenteral colistin, both for treating severe healthcare-associated infections and as part of surgical prophylaxis regimens involving selective digestive decontamination [11].

In contrast to these relatively restricted human indications, colistin has seen far more extensive application in veterinary medicine, particularly in intensive animal production systems. For decades, it has been employed to treat and prevent enteric infections caused by *E. coli* in poultry and swine, and historically was even administered as a growth promotor [17]. Colistin is additionally administered to laying hens as well as to cattle, sheep, and goats that produce milk intended for human consumption [11]. However, with increasing concern about preserving the effectiveness of colistin against multidrug-resistant human pathogens and the potential for animal-to-human transmission of colistin-resistant bacteria, its veterinary use has come under critical reevaluation and tighter regulation in recent years [11].

In 2006, the European Union (EU) took its first steps to regulate colistin use in animals by prohibiting its inclusion in feed for growth promotion purposes [18]. A decade later, in 2016, the European Medicines Agency (EMA) recommended that countries within the EU with high levels of polymyxin use in livestock should aim to lower consumption to less than 5 mg/PCU (per population correction unit) by 2020 [11]. Despite these efforts, EMA surveillance data from 2022 revealed that six member states—Croatia, Cyprus, Germany, Hungary, Lithuania, and Poland—had not yet met this target [19]. Between 2011 and 2018 the sales of polymyxins in animals declined by nearly 70% in the EU [20]. Nevertheless, in 2017, the use of colistin remained substantially higher in food-producing animals than in humans, with estimated consumption at 3.2 mg/kg of animal biomass compared to 0.06 mg/kg in the human population [20]. Most recently, the 2023 EMA’s report indicated that average polymyxin sales across 29 reporting countries reached 1.2 mg/kg of animal biomass, accounting for 2.7% of the total volume of veterinary antimicrobials sold within the EU [21].

Recent works, such as the study by Shen et al. (2021) [22], have demonstrated that antimicrobial stewardship efforts aimed at lowering colistin consumption in food-producing animals can influence the occurrence of colistin-resistant strains in human intestinal colonization, highlighting the close relationship between antimicrobial use in veterinary and human medicine and reinforce the importance of integrated surveillance strategies under the One Health framework.

Concerning companion animals, reliable data on antimicrobial usage is currently limited and available primarily from select regions, such as the EU, where estimates are often based on veterinary antibiotic tablet sales reposts, which may not fully capture the real patterns of antimicrobial application in pets [19]. In companion animals, colistin is used in topical formulations—such as ear and eye drops, either alone or in combination with other antimicrobials [11]. By contrast, oral colistin in tablets are used in calves to prevent and treat neonatal colibacillosis [11]. Even in regions where monitoring systems are in place, determining the exact usage patterns of HPCIAs across different pet species remains a significant challenge. An European cross-sectional study involving Belgium, Italy, and the Netherlands reported that polymyxins constituted approximately 6% of the antimicrobial treatments in pets [23]. Complementary findings from Germany showed polymyxin usage in dogs and cats at a single veterinary hospital in 2017 and 2018 accounted for just 0.06% and 0.13% of total antibiotic use, respectively [24]. In Japan, companion animal clinics reportedly used around 29.9 tons of antimicrobials during 2017–2018, with colistin making up less than 1% of this amount [25]. Access to this information is essential to identify the clinical contexts that may benefit from targeted stewardship strategies as, despite low usage, the close contact between companion animals and humans raises concerns about companion animals serving as reservoirs for colistin-resistant bacteria.

## 3. Colistin Resistance

Polymyxins, as cationic antimicrobial peptides, exert their bactericidal effect primarily through electrostatic interactions with the negatively charged outer membrane of Gram-negative bacteria. They exhibit high affinity for the lipid A portion of lipopolysaccharide (LPS), displacing divalent cations such as magnesium (Mg^2+^) and calcium (Ca^2+^) from phosphate groups on the LPS. This displacement destabilizes the outer membrane, compromises its integrity, and ultimately results in leakage of cellular contents and bacterial cell death [26].

While the full molecular basis of polymyxin resistance is not yet fully understood, it is well established that modifications to the LPS are a major resistance mechanism in Gram-negative organisms, particularly in relation to colistin. These modifications typically involve the addition of positively charged groups such as phosphoethanolamine (pEtN) and/or 4-amino-4-deoxy-L-arabinose (L-Ara4N) to the lipid A moiety.

### 3.1. Intrinsic Resistance Mechanisms

Some Gram-negative bacteria exhibit intrinsic resistance to polymyxins, which is typically driven by the constitutive expression of genes responsible for modifications of the LPS layer. In species such as *Proteus mirabilis* and *Serratia marcescens*, resistance has been linked to the ongoing activity of the *arnBCADTEF* operon and/or the *eptB* gene. These genetic determinants mediate the covalent addition of L-Ara4N and/or pEtN to the lipid A portion of the LPS [27]. This alteration reduces the net negative charge of the bacterial outer membrane, thereby weakening the electrostatic interaction between LPS and the positively charged polymyxin molecules. As a consequence, these organisms exhibit reduced susceptibility to polymyxins even in the absence of selective pressure [7,27].

### 3.2. Acquired Resistance Mechanisms

Acquired resistance to polymyxins in Gram-negative bacteria often results from structural alterations in the bacterial cell envelope, primarily involving LPS modifications. These changes are typically mediated by mutations in regulatory genes or by the acquisition of MGEs [27].

#### 3.2.1. PmrAB Two-Component System

**Figure 1:**
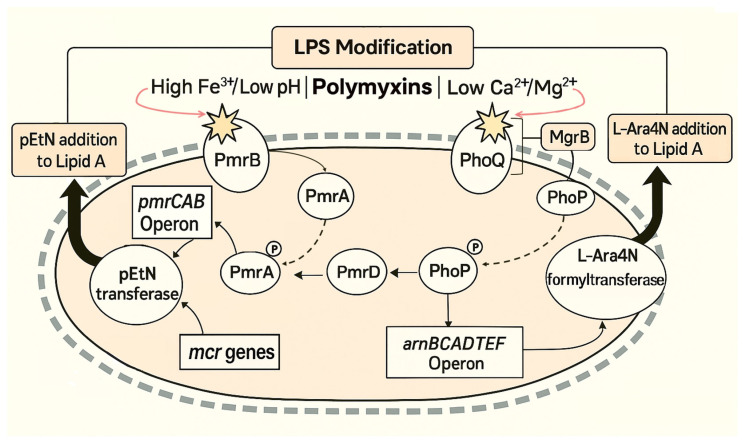
Schematic overview of lipopolysaccharide (LPS) modification pathways contributing to polymyxin resistance in Gram-negative bacteria. Gram-negative bacteria can detect a variety of environmental signals—such as exposure to polymyxins and other cationic antimicrobial peptides, low Ca^2+^ or Mg^2+^ availability, high Fe^3+^ concentrations, or acidic pH—which stimulate the histidine kinases PmrB and PhoQ (denoted by star symbols). Phosphorylation-dependent activation steps are indicated with dashed arrows, while thick arrows illustrate the downstream effects. Signal transduction through the PmrA–PmrB and PhoP–PhoQ two-component systems triggers expression of the *pmrCAB* and *arnBCADTEF* operons, respectively. The *pmrCAB* encodes enzymes that attach pEtN to lipid A, while *arnBCADTEF* operon mediates the synthesis and transfer of 4-amino-4-deoxy-L-arabinose (L-Ara4N) to lipid A. These chemical modifications, carried out by pEtN transferase and L-Ara4N formyltransferase (thick arrows), reduce the binding affinity of colistin for the bacterial membrane. Loss-of-function mutations in *mgrB*, a negative regulator of PhoP–PhoQ, abolish feedback inhibition, causing constitutive *phoP–phoQ* expression and overproduction of L-Ara4N. In addition, plasmid-borne *mcr* genes encode pEtN transferases that replicate the chromosomal pEtN modification pathway but enable its rapid dissemination between bacterial species via horizontal gene transfer.

One well-characterized mechanism involves mutations in the *pmrA* and *pmrB* genes, which encode the PmrAB two-component regulatory system. Under certain environmental conditions—such as the presence of cationic compounds like polymyxins, high Fe^3+^ concentration or acidic pH—PmrB phosphorylates PmrA, which in turn induces the expression of the *pmrCAB* operon and the *arnBCADTEF* (also known as *pmrHFIJKLM*) operon, both of which contribute to the LPS modification (Figure 1), decreasing its net negative charge and reducing polymyxin binding [27,28]. Mutations in this system have been associated with colistin resistance in several clinically relevant species, including *E. coli* [29], *Enterobacter aerogenes* [30], *Klebsiella pneumoniae* [31], *Salmonella enterica* [29] and *P. aeruginosa* [32].

#### 3.2.2. PhoPQ Two-Component System

The PhoPQ system plays a parallel role in polymyxin resistance. It also responds to the presence of antimicrobial peptides, acidic pH and low Ca^2+^ or Mg^2+^ levels [27]. PhoQ, a membrane-bound sensor kinase, is activated by these stressors and phosphorylates PhoP, the cytoplasmic response regulator. Activated PhoP induces the expression of *arnBCADTEF* operon, promoting L-Ara4N addition to lipid A (Figure 1). Moreover, PhoP can activate the PmrAB system indirectly via the PmrD connector protein, further enhancing LPS modification through pEtN addition [27,33,34].

Inactivation or mutation of the *mgrB* gene, which codes the small regulatory protein MgrB that negatively controls PhoPQ activity, leads to the PhoPQ system’s constitutive activation due to *phoPQ*-dependent genes remaining upregulated, thus contributing to persistent resistance [31,35]. Such mutations are commonly found in colistin-resistant *K. pneumoniae* strains [35,36] and have also been described in *E. coli* strains [37].

#### 3.2.3. Plasmid-Mediated Colistin Resistance

In addition to chromosomally encoded resistance mechanisms, the horizontal transfer of plasmid-mediated *mcr* (mobilized colistin resistance) genes—most notably *mcr-1*, first reported in 2015 in *E. coli* isolates from food-producing animals in China and associated with an IncI2-type plasmid [38]—has emerged as a major public health concern. These genes encode a pEtN transferase that catalyzes the addition of pEtN to lipid A (Figure 1), thereby mimicking chromosomal resistance mechanisms but enabling horizontal gene transfer [9,38]. The expression of *mcr* genes alone is sufficient to confer phenotypic resistance to colistin in various Gram-negative bacteria, particularly in members of the Enterobacterales [9,39].

Since the initial discovery of *mcr-1*, global surveillance initiatives have identified additional *mcr* homologs, expanding the *mcr* gene family to ten distinct variants (Table 1) with more than 100 allelic variants currently deposited in public databases such as GenBank [40]. These *mcr* genes were identified across different geographic regions, many of them associated with conjugative, transmissible plasmids (Table 1), and have been detected in a broad range of bacterial species isolated not only from livestock but also from humans [13,14], food products [41,42], companion animals (Table 2), wild animals [43,44,45] and environmental sources [13]. Their presence across such diverse reservoirs highlights the potential for rapid interhost dissemination via horizontal gene transfer [38,46]. This has raised significant global public health concerns, as colistin is considered a last-resort antibiotic for treating infections caused by MDR Gram-negative bacteria [11,39].

Recent evidence has identified *mcr-1* in *E. coli* isolates dating back to the early 1980s, indicating that this resistance gene emerged earlier than previously recognized [56]. Phylogenetic analyses suggest that all currently circulating *mcr-1* mobile elements descend from a single mobilization event in the mid-2000s, mediated by a composite transposon formed of a ~2600 bp region containing the *mcr-1* gene (1626 bp) and a putative open reading frame encoding a PAP2 superfamily protein (765 bp), flanked by two IS*Apl1* insertion sequences (IS*Apl1*–*mcr-1*–PAP2–IS*Apl1*). This was followed by a rapid demographic expansion and global dissemination [57]. Subsequent loss of flanking IS*Apl1*, due to its inherent instability, is thought to have stabilized the *mcr-1* gene within diverse plasmid backbones, thereby facilitating its long-term persistence and global spread across bacterial populations [57].

To date, *mcr* genes have been identified on a wide variety of plasmid backbones, and in companion animals these include IncX4, IncI2, IncHI2, IncFIB, IncP1 and also IncFIA/FII(K) hybrid plasmids (Table 2). This diversity suggests that, even in settings where colistin use is restricted, such as in veterinary medicine, plasmid-mediated colistin resistance may persist and disseminate through pet-associated bacterial populations.

**Table 2:** Overview of *mcr*-carrying isolates from companion animals across the world.

Region	Country	Year	Host	Source	Bacterial Species	Mobile Genetic Elements	Other Antibiotic Resistance Genes in the Same Plasmid	Ref.
*mcr-1*								
Africa	Algeria	2025	Cat	Commensal (rectal swab)	*Enterobacter kobei*	N/A	N/A	[58]
Asia	China	2015	Cat	Infection (diarrhea)	*Escherichia coli*	IncX3-X4 plasmid, IS*Aba125*	*bla*_NDM-5_	[5]
	China	2016	Dog and Cat	Commensal (feces)	*Escherichia coli*	N/A	N/A	[15]
	China	2016	Cat	Commensal (rectal swab)	*Escherichia coli*	IncX3-X4 plasmid, IS*Aba125*	*bla*_NDM-5_	[4]
	China	2017	Dog	Commensal (rectal swabs)	*Escherichia coli*	IncI2, IncX4 and IncHI2 plasmids, IS*Apl1*	*bla*_CTX-M-14_	[59]
	China	2017	Dog	Commensal (feces)	*Escherichia coli*	IncX4-like plasmid, IS*Ecp1*	*bla*_CTX-M-55_	[60]
	China	2017	Dog and Cat	Commensal (nasal and rectal swabs)	*Escherichia coli* and *Klebsiella pneumoniae*	N/A	N/A	[61]
	China	2018	Dog and Cat	Commensal (feces) and Infection (urine, nasal secretion, diarrhea)	*Escherichia coli*	IncHI2 plasmid and IS*Apl1*, IncI2	*bla*_CTX-M-14_, *bla*_CTX-M-64_, *floR* and *fosA3*	[62]
	China	2018	Cat	Infection (tracheal lavage)	*Klebsiella pneumoniae*	IncX4 plasmid, IS*26*	None	[63]
	China	2019	Dog	Infection	*Escherichia coli*	IncI2 plasmid	*bla*_CTX-M-55_	[64]
	China	2020	Dog	Commensal (feces)	*Escherichia coli*	IncX4 plasmid, IS26	None	[65]
	China	2021	Dog	Commensal (feces)	*Klebsiella pneumoniae*	N/A	N/A	[66]
	South Korea	2020	Dog	Infection (diarrhea)	*Escherichia coli*	IncI2 plasmid	None	[67]
	Taiwan	2019	Dog	Infection (UTI)	*Enterobacter cloacae* and *Klebsiella pneumoniae*	None	N/A	[68]
Europe	France	2019–2020	Dog and Cat	Commensal (feces)	*Escherichia coli*, *Rahnella aquatili*	N/A	N/A	[69]
	Germany	2011	Dog and Cat	Commensal (feces)	*Escherichia coli*	IncX4 plasmid	None	[70]
	Portugal	2018	Dog	Commensal (feces)	*Escherichia coli*	IncHI2A plasmid	*sul1*, *dfrA1*, *aadA1*	[14]
South America	Argentina	2019	Dog	Infection (UTI)	*Escherichia coli*	IncI2 plasmid	None	[71]
	Brazil	2020	Dog	Infection (UTI, abdominal seroma, nasal secretion)	*Escherichia coli*, *Klebsiella* spp., *Enterobacter* spp.	N/A	N/A	[72]
	Brazil	2021	Cat	Infection (UTI)	*Klebsiella pneumoniae*	N/A	N/A	[73]
	Ecuador	2016	Dog	Commensal (rectal swab)	*Escherichia coli*	IncI2	None	[74]
	Ecuador	2019	Dog	Commensal (feces)	*Escherichia coli*	N/A	N/A	[75]
*mcr-2*								
Asia	China	2019	Dog	Commensal (feces)	*Klebsiella pneumoniae*	N/A	N/A	[66]
*mcr-3*								
Asia	China	2019	Dog	Commensal (feces)	*Klebsiella pneumoniae*	N/A	N/A	[66]
	China	2020	Dog	Commensal (feces)	*Escherichia coli*	IncP1 plasmid, TnAs2, IS26	None	[65]
	Taiwan	2021	Dog	Infection	*Escherichia coli*	N/A	N/A	[76]
*mcr-4*								
Asia	China	2019	Dog	Commensal (feces)	*Klebsiella pneumoniae*	N/A	N/A	[66]
*mcr-5*								
Asia	China	2019	Dog	Commensal (feces)	*Klebsiella pneumoniae*	N/A	N/A	[66]
*mcr-8*								
Asia	China	2017	Cat	Infection (UTI)	*Klebsiella pneumoniae*	IncFIA (HI1)/FII(K) plasmid, IS*Ecl1,* IS*Kpn26*	None	[77]
*mcr-9*								
Africa	Egypt	2017	Dog and Cat	Infection (ocular swab, nasal swab)	*Enterobacter hormaechei*	IncHI2	*bla*_VIM-4_	[78]
Asia	China	2019	Dog	Commensal (feces)	*Klebsiella pneumoniae*	N/A	N/A	[66]
	Japan	2021	Cat	Infection (nasal cavity swab)	*Enterobacter asburiae*	IncHI2 plasmid	*aac(6′)-Ib3*, *aph(6)-Id*, *bla*_TEM-1B_, *dfrA19*, *aac(6′)-Ib-cr*, *catA2*, *tetD*	[79]
	Thailand	2022	Cat	Infection (abdominal fluid)	*Enterobacter hormaechei*	IncHI2/2A plasmid	N/A	[80]
Europe	UK	2021	Dog	Infection (SSTI)	*Escherichia coli*	N/A	N/A	[81]
*mcr-10*								
Asia	China	2019	Dog	Infection	*Klebsiella pneumoniae*	N/A	N/A	[66]
	Japan	2021	Dog	Infection (pus)	*Enterobacter roggenkampii*	IncFIB plasmid	None	[82]

N/A, Not applicable; SSTI, skin and soft tissue infection; UTI, urinary tract infection.

## 4. Methods for Colistin Susceptibility Testing

### 4.1. Challenges and Technical Limitations

Although colistin has been used in clinical practice for several decades, establishing a standardized and reliable method for susceptibility testing has proven challenging. This difficulty is related to multiple factors, including its poor diffusion into agar, the cationic properties of polymyxins leading to adherence to laboratory plastics, the occurrence of heteroresistance (a phenomenon in which a bacterial population contains both susceptible and resistant subpopulations to a given antibiotic, leading to inconsistent or unstable resistance expression), and the absence of a reliable reference method for comparisons [83,84]. As result, technical complications frequently arise, which can lead to inaccurate susceptibility determinations. A survey conducted in 2017 found that many laboratories either did not perform colistin susceptibility testing on-site or relied on methods that are not recommended [85].

The accuracy of susceptibility testing is strongly affected by external variables. For instance, polymyxin resistance is regulated by the two-component systems PhoP/PhoQ and PmrA/PmrB, which are activated by fluctuations in pH and divalent cation concentrations such as calcium, magnesium, and iron [27]. Considerable variability in the cation content of Mueller-Hinton broth (MHB) has been reported across commercial brands, and calcium and magnesium levels are frequently lower than the Clinical and Laboratory Standards Institute (CLSI) specifications [86]. This variation may result in misinterpretation of minimum inhibitory concentrations (MICs). Accordingly, CLSI suggests employing cation-adjusted MHB or enriching the culture medium with cations for colistin susceptibility testing [87,88].

However, it has also been demonstrated that MICs obtained with cation-adjusted MHB, as recommended by CLSI, may be significantly distorted—showing falsely elevated susceptibility in *P. aeruginosa* and *A. baumannii*, while underestimating MICs for *E. coli* strains [89]. Notably, the calcium concentration prescribed by CLSI for in vitro testing is approximately twice the one found in human interstitial fluid [89]. Consequently, there is still no consensus on whether cation-adjusted or non-adjusted MHB should be considered the standard medium in colistin susceptibility testing.

Furthermore, resistance may be unstable, as demonstrated by studies showing loss of colistin resistance after subculture without selective pressure [90,91,92], or after long-term storage at −70 °C [84].

### 4.2. Reference Methods: Broth Microdilution

Currently, broth microdilution (BMD) is the only method endorsed by CLSI and EUCAST (European Committee on Antimicrobial Susceptibility Testing) for reliable determination of colistin susceptibility testing [93]. According to CLSI guidelines, BMD should be performed using cation-adjusted MHB, with two-fold dilutions of colistin ranging from 0.12 to 512 µg/mL and a final bacterial inoculum of 5 × 10^5^ CFU/mL per well [94]. Despite being the gold standard, BMD is technically demanding and labor-intensive, particularly in laboratories without automated platforms. In addition, the method is prone to variability, including the occurrence of “skip wells” [95]. This term refers to an unusual growth pattern in microdilution assays, in which bacterial growth is absent at a given antibiotic concentration but reappears at higher concentrations, leading to irregular MIC readings. Such phenomena have been documented in *Enterobacter* spp. [83], linked to heteroresistant subpopulations [83,96]. In *P. aeruginosa* strains, “skip wells” have been linked to increased expression of the *pmrAB*, *phoQ*, and *arn* genes, which mediate structural modifications of LPS, thereby reducing available binding sites for polymyxins [97]. Nonetheless, BMD remains the gold standard owing to its reproducibility, reliability, and adaptability to automation.

Alternative dilution-based approaches have also been evaluated. The broth macrodilution method, which employs test tubes instead of microtiter plates, is conceptually identical to BMD and has shown strong agreement, with no false susceptibility results when compared with the reference method [84]. However, its laborious nature and lower throughput limits its practical use. Agar dilution has also been widely employed in research settings and occasionally implemented in commercial formats [98,99]. Although it is included in CLSI protocols [87], the joint CLSI-EUCAST Polymyxin Breakpoints Working Group has explicitly stated that agar dilution is not recommended for colistin susceptibility testing [93]. More broadly, agar-based approaches—including agar dilution, gradient diffusion, and disk diffusion—are particularly unreliable due to the poor diffusion of colistin into agar matrices and have consistently proven inaccurate for assessing polymyxin susceptibility [100].

#### Commercial Microdilution Systems

Several commercial microdilution assays have been developed to facilitate routine testing. These systems differ in their format, concentration ranges, level of automation, and reported performance compared with BMD. While most are user-friendly and provide acceptable reproducibility, their accuracy varies substantially depending on the bacterial species tested and the resistance mechanism involved, particularly in the cases of heteroresistance or presence of plasmid-mediated *mcr* genes:

- BD Phoenix: This platform allows for manual or automated inoculation and tests colistin concentrations from 0.5 to 4 µg/mL, with turnaround times of 6–16 h. While it reliably detects plasmid-mediated colistin resistance, BD Phoenix (BD Diagnostics, Franklin Lakes, NJ, USA) has a high false-susceptible rate (~15%) and shows limited ability to identify heteroresistant populations [95].
- MicroScan: Requires manual inoculation, incubation for 16–20 h and has a narrow MIC range (2–4 µg/mL) [101]. MicroScan (Beckman Coulter, Brea, CA, USA) reported performance varies substantially by species, with high categorical agreement for Enterobacterales (99.3%) but poor for non-fermenting Gram-negative bacilli (64.1%). A high rate of major errors (26.9%) was reported, mostly due to MIC overestimation in non-fermenters [102,103].
- Sensititre: Sensititre (Thermo Fisher, Waltham, MA, USA) features a wide MIC range of 0.12–128 µg/mL and incubation times of 18–24 h, with inoculation possible either manually or via autoinoculator. In one study, the system achieved 96% categorical agreement without false susceptibility, representing the most reliable performance among commercial microdilution platforms [84].
- UMIC: The UMIC Colistine kit (Biocentric, Bandol, France) is a manual-based system designed for individual isolate testing. This system covers a MIC range from 0.0625 to 64 µg/mL, with a required incubation time of 18–24 h. Studies indicate good reproducibility and high categorical agreement, with 92.5% for Enterobacterales and 89.7% for non-fermenting Gram-negative bacteria, while essential agreement ranges from 94–100% for Enterobacterales but may fall below 80% for non-fermenters [103,104,105].
- VITEK2: The system is fully automated and provides rapid results within 4–10 h, testing colistin concentrations from 0.5 to 16 µg/mL. VITEK2 (bioMérieux, Marcy-l’Étoile, France) shows poor sensitivity for detecting resistant strains and heteroresistant subpopulations, leading to false susceptibility readings [106,107].

### 4.3. Molecular Approaches for Resistance Detection

Molecular methods provide valuable complementary tools for detecting known resistance genes or mutations, but they require further optimization and cannot serve as standalone susceptibility assays. While these techniques can identify known resistance determinants, like the *mcr* genes [94], the absence of such markers does not confirm susceptibility, nor do they yield MIC resistance values. Moreover, many chromosomal determinants of resistance remain unknown, the functional consequences of certain mutations in the LPS biosynthesis pathways are often uncertain, and resistance levels may also be affected by variable gene expression, limiting their routine application [95]. Examples of molecular approaches include:

- Conventional PCR: Standard PCR assays allow detection of individual *mcr* genes (simplex) or multiple variants in the same reaction (multiplex). Primer sets have been published for *mcr-1* through *mcr-5* [108] and *mcr-6* to *mcr-9* [109], enabling specific detection directly from bacterial isolates. Most recently a tenfold multiplex PCR method for *mcr-1* to *mcr-10* was developed showing a high specificity [110]. Results can generally be obtained within the same working day. These assays are considered reference methods for validating novel molecular tools.
- Real-Time PCR (qPCR): Several quantitative PCR assays have been developed to detect *mcr* genes directly from cultured bacteria, clinical samples, or stools. An SYBR Green-based assay demonstrated 100% specificity and a limit of detection of 10^2^ CFU, with no false-positive results. Importantly, the assay was also conclusive when applied to stool samples spiked with *mcr-1*-positive *E. coli* [111]. Similarly, a TaqMan probe-based qPCR with a detection range of 10^1^–10^8^ DNA copies achieved 100% specificity when applied to bacterial isolates and fecal samples from chickens [112]. More recently, a multiplex TaqMan real-time PCR assay was introduced for the simultaneous detection of *mcr-1* to *mcr-10*, offering high specificity, sensitivity, and reproducibility, and thus representing a powerful tool for comprehensive resistance surveillance [113].
- Whole Genome Sequencing (WGS): Screens the entire bacterial genome, identifying plasmid-mediated *mcr* genes and chromosomal mutations. Specificity approaches 100%, with a turnaround time of 1–2 days, depending on sequencing platform. WGS also enables high-resolution epidemiological typing but requires bioinformatics expertise and higher costs [108,109,114].
- Loop-Mediated Isothermal Amplification (LAMP): The *eazyplex^®^ SuperBug* kit (Amplex Biosystems GmbH, Giessen, Germany) detected *mcr-1* with 100% sensitivity and specificity, delivering results in ~20 min. However, the system is limited to six samples per hour, and has not been validated for direct detection without pre-culture [115]. A multiplex LAMP assay later expanded detection to *mcr-1* through *mcr-5*, also achieving 100% accuracy [116].
- DNA microarrays: Microarray-based assays enable parallel detection of numerous resistance determinants. The commercial Check-MDR CT103XL system (Check-Points Health, Wageningen, The Netherlands) can simultaneously detect *mcr-1* and *mcr-2* genes along with a wide range of β-lactamases encoding genes directly from Enterobacterales cultures. Results are available in approximately 6.5 h, with reported 100% sensitivity and specificity [117]. While highly powerful for surveillance, the method remains costly and technically complex, which limits its applicability for routine clinical diagnostics.

### 4.4. Novel and Emerging Assays

Recent innovations aim to provide rapid and accurate alternatives to standard methods, most of which are qualitative in nature:

- Rapid Polymyxin NP test: This colorimetric assay detects resistance based on glucose metabolism in the presence of colistin. It has shown specificity and sensitivity of 99.3% and 95.4%, respectively, compared to BMD [118]. Importantly, it can identify heteroresistant populations and plasmid-mediated MCR-1 producers. The commercial version (Rapid Polymyxin NP test; ELITechGroup Microbiology, Puteaux, France) provides results within 2–3 h, making it suitable for routine diagnostics.
- Lateral flow immunoassay (LFIA): Monoclonal antibody-based LFIA (NG Biotech, Guipry, France) enables rapid detection of MCR-1-producing isolates directly from bacterial colonies. It shows 100% sensitivity and 98% specificity, but does not detect other producers of other MCR-variants [119]. Its speed (<15 min), low cost, and simplicity make it highly attractive for implementation in clinical and veterinary microbiology laboratories.
- Micromax technology: The Micromax assay (Halotech DNA SL, Madrid, Spain) is based on detection of DNA release following cell wall damage in the presence of colistin. It demonstrated 100% sensitivity and 96% specificity in *A. baumannii*, with results obtained within 3.5 h [120]. However, its technical complexity and cost currently limit widespread use.

## 5. Epidemiology of Colistin Resistance in Companion Animals

The global epidemiology of colistin resistance is still incompletely understood, largely because most laboratories continue to rely on inadequate testing methods, as highlighted in Section 4. Until reliable laboratory capacities become widely available, surveillance data will remain limited and fragmented. To address this gap, structured monitoring programs have been established in some regions. In Europe, the ECDC included colistin in its surveillance panel for carbapenem- and/or colistin-resistant *Enterobacteriaceae* (CCRE survey) coming from human clinical strains in 2019 [121], and since 2014, susceptibility testing for colistin in bacteria from food-producing animals has been mandatory under EU legislation [122]. These initiatives have provided critical insights, as livestock—particularly pigs—are recognized as key reservoirs for *mcr*-positive bacteria.

In companion animals, however, the emergence of colistin resistance has received far less attention, with only a handful of national programs explicitly addressing antibiotic consumption and resistance monitoring in dogs and cats. Available information relies heavily on independent studies reporting resistant bacteria in both infected and colonized dogs and cats, with most focusing on *mcr* gene detection. The recently established European Antimicrobial Resistance Surveillance Network in Veterinary Medicine (EARS-Vet) represents a first step toward coordinated monitoring in companion animals [123]. However, no reports have yet been released, and it remains unclear whether colistin is systematically included in their testing panels.

Some national surveillance systems have nonetheless provided valuable insights. In Switzerland, the national resistance monitoring program [124] reported colistin-resistant *E. coli* from clinical urine submissions of dogs and cats, as well as *P. aeruginosa* from canine otitis, although the prevalence remained ≤1%. Importantly, all resistant isolates lacked known *mcr* genes, underscoring the role of alternative resistance mechanisms [124]. Likewise, in France, longitudinal AMR monitoring by the RESAPATH network [125] revealed a significant decline in colistin resistance among *E. coli* isolates from both dogs and cats between 2012 and 2022, paralleling reductions observed in livestock. In China, a particularly illustrative example of how policy interventions can influence resistance trends has been reported. Following the national ban on colistin as a growth promoter in 2017, data from the China Antimicrobial Resistance Surveillance Network for Pets (CARPet) indicated a clear decline in colistin-resistant *E. coli* and *mcr-1*-positive strains from dogs and cats compared to pre-ban years [126]. These examples demonstrate that national surveillance programs can yield valuable insights into the epidemiology of colistin resistance in companion animals.

Furthermore, *mcr* genes have been detected in dogs and cats from at least eleven countries, illustrating the widespread and multifaceted presence of *mcr* genes in companion animals. The *mcr-1* gene is by far the most frequently reported, followed by *mcr-9* which has been sporadically identified in the UK, Egypt, and Thailand (Table 2). All other known *mcr* variants have also been detected in companion animals, apart from *mcr-6* and *mcr-7*. China accounts for the largest number of reports, documenting a broad spectrum of variants involving primarily *E. coli* but also *K. pneumoniae*, and less common species such as *R. aquatili* and *E. asburiae* (Table 2).

Collectively, these studies span both clinical and commensal isolates and consistently demonstrate the diverse epidemiological context in which *mcr* genes occur. For instance, in Germany, *mcr-1*-positive *E. coli* ST10 strain was isolated from a barn dog cohabiting with pigs [70]; in Ecuador, an *mcr-1*-positive strain was found in dog feces in a public park [75]; and in Brazil, diverse infections in pets have yielded *mcr-1*-positive Enterobacterales [72,73].

Interestingly, *mcr*-positive strains from dogs and cats are often recovered from fecal or urinary samples (Table 2), even though colistin is rarely used orally or for the treatment of urinary tract infections in these species. This observation suggests that colonization or infection in companion animals may not necessarily result from direct antimicrobial pressure, but rather from contact with contaminated environments or other colonized hosts. This interpretation is further supported by surveillance data showing that, following the ban on colistin as a growth promoter in livestock, a parallel decline in colistin resistance has been observed in companion animals [126], as mentioned above.

The co-occurrence of multiple *mcr* genes in the same strain has also been described. In China, a dog-derived *E. coli* strain carried both *mcr-1.1* and *mcr-3.7* genes [65]. In this strain, the two resistance determinants were carried on separate plasmid backbones. The *mcr-1.1* gene was incorporated into an IncX4 plasmid, arranged in an IS26–*parA*–*mcr-1.1*–*pap2* cassette, while *mcr-3.7* was embedded within an IncP1 plasmid displaying the configuration Tn*As2*–*mcr-3.7*–*dgk*A–IS26 [65]. Similarly, combinations such as *mcr-1*/*mcr-3* and *mcr-1*/*mcr-5* have been identified in *K. pneumoniae* from companion animals [66]. This phenomenon is concerning as it may facilitate the dissemination of multiple *mcr* genes. Moreover, plasmids carrying *mcr* genes often harbor additional resistance determinants, including those conferring resistance to clinically important antibiotics in human medicine, such as third-generation cephalosporins (like *bla*_CTX-M-type_ genes) and carbapenems (like *bla*_NDM-5_ genes) (Table 2). This facilitates co-selection, whereby the use of antimicrobials other than colistin contributes to the persistence and spread of multidrug-resistant plasmids [127,128].

Recent evidence also indicates chromosomal integration of *mcr-1* in *E. coli*, *K. pneumoniae*, and *Enterobacter cloacae*, sometimes in the absence of flanking IS*Apl1* [68,126,129]. Such integration likely confers enhanced genetic stability, as the gene becomes part of the bacterial chromosome, reducing the likelihood of loss compared with plasmid-borne copies and potentially promoting long-term persistence of colistin resistance.

Taken together, these findings emphasize the underestimated role of companion animals in the dissemination of colistin resistance and reinforce the need for integrated surveillance programs that explicitly include pets within the One Health framework.

## 6. Transmission Potential and Dissemination Pathways

### 6.1. Companion Animals as a Reservoir for AMR Transmission

Europe is home to an estimated 352 million companion animals, distributed across approximately 166 million households, meaning that about half of all European households have at least one pet. Among these, 25% of households own a dog, while 27% have a cat [130].

Although pet ownership has well-recognized benefits for human physical and mental health [131], close human–animal interactions also contribute to the transmission and dissemination of AMR. Several studies have reported that pathogenic microorganisms and resistance genes can be shared between companion animals and cohabiting human [14,60,132,133,134].

**Figure 2:**
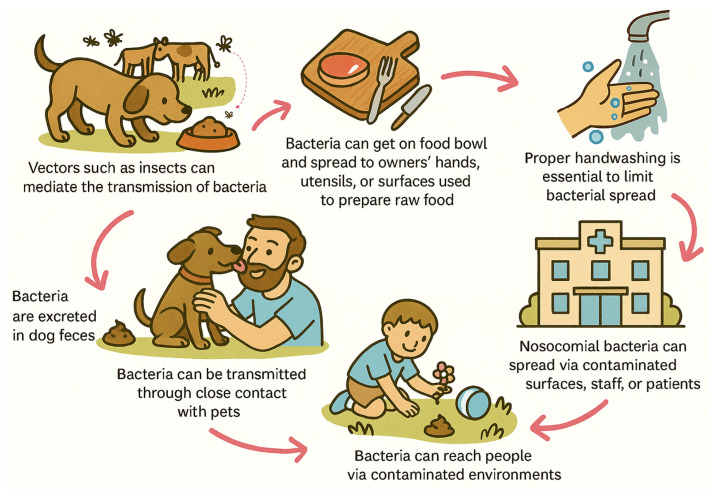
Potential routes of bacterial transfer between companion animals and humans.

Direct contact, such as petting, touching, or kissing, facilitates bacterial exchange (Figure 2), as pets exhibit grooming behaviors that promote colonization of their fur, skin, and oral cavity with AMR bacteria [135,136]. Surveys in the UK, Germany, and Belgium have shown that such intense contacts are common among pet owners, creating opportunities for AMR dissemination [137,138,139]. Indirect exposure can also occur through environmental contamination, since companion animals often use litter boxes or defecate in public spaces such as gardens, parks, and sidewalks, contaminating shared environments [135,136]. In addition to pet owners, veterinary personnel, students, and trainees represent professional risk groups frequently exposed to resistant strains through similar routes within the healthcare space [132,133].

Importantly, companion animals should not be considered in a vacuum, but as a part of a large ecosystem [60]. Environmental vectors such as flies and wild birds can act as additional links in AMR dissemination chains (Figure 2). In a longitudinal investigation of four commercial farms in China, colistin- and carbapenem-resistant *E. coli* were found in food-producing animals, dogs, flies, wild bird nests, sewage, and humans working on the farms, with similar plasmid backbones carrying resistance genes detected [60]. This study illustrates how dogs are in conjunction with other vectors reservoirs of antimicrobial resistance, reinforcing the need to consider companion animals as part of a broader ecological network of AMR dissemination.

### 6.2. Evidence of Interhost Transmission of Resistant Bacteria

Initially, colistin-resistant strains were thought to be disseminated primarily via food-producing animals through meat, milk, or eggs, or via direct livestock contact [13,140]. However, growing concern now points to interhost transmission within domestic settings. EMA, through its Antimicrobial Working Party reflection paper, has highlighted colistin-resistant *E. coli* as relevant pathogens that can be exchanged between companion animals and humans [141].

This concern is supported by a growing body of evidence. In one study conducted in China, households in which humans carried *mcr-1*-positive isolates were significantly more likely to also have dogs carrying *mcr-1*-positive bacteria, and vice versa [59]. Clonal relatedness of *mcr-1*-positive *E. coli* strains from dogs, cats, and one pet owner in Beijing, as determined by pulsed field gel electrophoresis (PFGE), further supports direct sharing, with pet food also identified as a possible source of transmission [61]. In another report, identical PFGE profiles were observed between *mcr-1*-positive *E. coli* ST354 strains from a dog and a human working in the same pet shop [15]. Similar findings were reported in Portugal, where WGS analysis confirmed the sharing of *mcr-1*-positive *E. coli* ST744 strains between dogs and cohabiting humans [14].

Although these findings clearly indicate bacterial sharing between humans and companion animals, it remains challenging to determine the exact direction of transmission.

### 6.3. Plasmid-Mediated Dissemination of Resistance Genes

The spread of AMR is primarily driven by the transfer of resistance genes through MGEs, including plasmids and transposons [142]. Although pathways of horizontal gene transfer are complex and often difficult to trace, accumulating evidence highlights the pivotal role of plasmids in colistin resistance dissemination.

For example, an IncX4 plasmid carrying *mcr-1* from a dog in China was nearly identical to plasmids from human isolates in Tanzania and pork samples in China, demonstrating cross-host and cross-regional dissemination [65]. Likewise, a large-scale study on commercial farms reported pHNSHP45-like plasmids, originally described in swine [38], across multiple reservoirs within the farms, including dogs, hatchery chickens, flies around poultry house, sewage, slaughterhouses, and even retail meat products from the same farms [60]. Moreover, this plasmid has also been detected in human isolates in other studies, further highlighting its broad dissemination potential [143,144].

In Ecuador, IncI2 plasmids carrying the *mcr-1.1* gene were found to be highly conserved across *E. coli* strains of different sequence types isolated from dogs, chickens, and humans living in the same household, supporting multi-host dissemination [74]. In Portugal, sharing of an MDR IncHI2A plasmid between a dog and its owner was reported [14]. This plasmid was also like one previously identified in an *E. coli* isolate from imported poultry meat originating from Italy [145]. Its MDR region carried *sul1*, *dfrA1*, and *aadA2* genes, conferring resistance to sulfonamides, trimethoprim, and aminoglycosides, respectively [14,145]. These findings suggest livestock-associated plasmids may spill over into companion animals, highlighting ecological link between agricultural antimicrobial use and resistance dynamics within the One Health continuum and the importance of controlling plasmid-mediated dissemination.

## 7. Strategies to Reduce Dissemination Risks

Mitigating the AMR transmission between companion animals and humans requires coordinated action at multiple levels within a One Health framework. Despite the increasing recognition of this issue, significant knowledge gaps persist, particularly regarding the prevalence of resistant pathogens in the pet population and the incidence of human infections directly attributable to companion animals. Addressing these shortcomings is essential to guide effective interventions.

Coordinated surveillance of zoonotic pathogens and antimicrobial resistance in household pets, combined with studies that estimate the burden of human disease and identify risk behaviors associated with interspecies transmission, is a crucial first step [134]. In parallel, education of pet owners, veterinarians, and healthcare professionals is needed to raise awareness about zoonotic risks, the importance of good hygiene practices, and the prudent use of antimicrobials [134,146,147]. Publicly accessible databases that integrate AMR and antimicrobial use data from companion animals would further strengthen transparency and inform decision-making.

Another central pillar is the implementation of antimicrobial stewardship programs in veterinary medicine, which remain limited compared with long-standing initiatives in human healthcare [148]. Such programs aim to promote prudent prescribing practices, strengthen infection control measures, and reduce the selective pressure favoring resistant strains. Specific prescribing guidelines tailored to companion animals represent valuable tools, but they must consider local resistance patterns to ensure efficacy [146,148]. Pet owners exert considerable influence on prescribing decisions; expectations of antibiotic therapy and the difficulty of interpreting animal symptoms often drive inappropriate prescriptions [146], highlighting the need for targeted communication and owner education.

While colistin use in companion animals is rare and often limited to topical formulations, co-selection driven by other antimicrobial classes—such as β-lactams, fluoroquinolones, and aminoglycosides—likely plays a key role in maintaining and disseminating *mcr*-carrying plasmids. Therefore, minimizing unnecessary antimicrobial exposure overall, rather than focusing solely on last-resort drugs, is a cornerstone strategy in limiting resistance emergence.

Equally important are infection prevention and environmental safeguards. All veterinary hospitals and clinics, regardless of size, should adopt infection prevention and control programs aimed at disrupting transmission routes and reducing nosocomial spread [149]. In addition, veterinary waste and effluents from pharmaceutical manufacturing, hospitals, and pet shops must be rigorously monitored, as their release into waterways contributes to the accumulation of clinically relevant resistance genes, such as *mcr*, in soil and aquatic environments [149,150]. Preventive measures, including vaccination of companion animals, can further reduce the need for antibiotic therapy by limiting the occurrence of bacterial and viral infections [134,151].

Finally, global coordination is critical. Given the transboundary movement of people, goods, and animals, antimicrobial resistance in companion animals cannot be effectively contained by regional policies alone. Comprehensive global data on antimicrobial use in pets, colonization dynamics, and plasmid genetics are essential to understand the international dissemination of colistin resistance and to guide evidence-based control measures.

## 8. Conclusions

Colistin resistance in companion animals, particularly dogs and cats, poses a substantial challenge to both veterinary and public health. Although colistin is not routinely used systemically in companion animals, being mainly applied topically, these animals can still harbor colistin-resistant bacteria. This suggests that resistance in pets may arise not primarily from direct antimicrobial use, but from spillover events associated with human, livestock, or environmental sources. The reduction of colistin use in livestock has been linked to lower colonization rates in food-producing animals; however, companion animals likely remain exposed through environmental contamination or close human contact.

Consequently, dogs and cats may serve as reservoirs or maintenance hosts of resistant bacteria and plasmids, even in the absence of direct antimicrobial selection pressure. Reports from Asia, Europe and South America further document household transmission, where humans and companion animals harbor closely related *mcr*-positive bacterial strains or plasmids, highlighting the real risk of direct transfer.

Addressing this issue requires a multifaceted approach that combines robust surveillance, responsible antimicrobial use, infection prevention, educational initiatives, and environmental monitoring. Currently, active surveillance in companion animals remains limited, despite their close contact with humans. Implementing comprehensive epidemiological monitoring, along with appropriate use of standardized susceptibility testing methods, is essential to accurately assess the prevalence and distribution of resistance. Restricting or prohibiting colistin use is often adopted as a strategy, based on the assumption that the expression of resistance genes carries a biological cost to bacteria, which could lead to their loss in the absence of selective pressure. However, the reversibility of resistance is not straightforward, as factors beyond selective pressure influence the stability and maintenance of resistance determinants within bacterial populations.

Ultimately, colistin resistance in companion animals must be interpreted through a One Health lens, as part of the interconnected human–animal–environment interface. Only through integrated, data-driven approaches can effective guidelines and interventions be developed to mitigate the spread of colistin resistance and preserve the efficacy of this last-resort antibiotic.

## Figures and Tables

**Table 1:** First reports of *mcr* genes across different hosts, countries, and bacterial species.

Gene	Plasmid Type	Year	Country	Host	Bacterial Species	Ref.
*mcr-1*	IncI2	2015	China	Pig	*Escherichia coli*	[38]
*mcr-2*	IncX4	2016	Belgium	Calves and pigs	*Escherichia coli*	[47]
*mcr-3*	IncHI2	2017	China	Pig	*Escherichia coli*	[48]
*mcr-4*	ColE	2017	Italy	Pig	*Salmonella enterica*	[49]
*mcr-5*	ColE	2017	Germany	Poultry	*Salmonella* Paratyphi B	[50]
*mcr-6*	IncX4	2017	UK	Pig	*Moraxella pluranimalium*	[51]
*mcr-7*	IncI2	2018	China	Chicken	*Klebsiella pneumoniae*	[52]
*mcr-8*	IncFII	2018	China	Pig	*Klebsiella pneumoniae*	[53]
*mcr-9*	IncHI2	2019	USA	Human	*Salmonella enterica*	[54]
*mcr-10*	IncFIA	2020	China	Human	*Enterobacter roggenkampii*	[55]

## Data Availability

No new data were created or analyzed in this study. Data sharing is not applicable to this article.

## Abbreviations

The following abbreviations are used in this manuscript:

AMR	Antimicrobial resistance
BMD	Broth microdilution
CLSI	Clinical and Laboratory Standards Institute
ECDC	European Centre for Disease Prevention and Control
EMA	European Medicines Agency
EU	European Union
EUCAST	European Committee on Antimicrobial Susceptibility Testing
HPCIA	Highest priority critically important antimicrobials
MDR	Multidrug-resistant
MGE	Mobile genetic elements
MHB	Mueller-Hinton broth
MIC	Minimum inhibitory concentration
L-Ara4N	4-amino-4-deoxy-L-arabinose
PDR	Pan-drug-resistant
pEtN	Phosphoethanolamine
UTI	Urinary tract infection
XDR	Extensively drug-resistant
